# Design and Optimization of High-G Graphene MEMS Acceleration Sensor

**DOI:** 10.3390/mi17080899

**Published:** 2026-07-27

**Authors:** Shengsheng Wei, Yina He, Yipeng Wang, Junqiang Wang, Mengwei Li

**Affiliations:** 1 Shanxi Key Laboratory of Graphene Sensing Materials and Devices, North University of China, Taiyuan 030051, China; 2 School of Semiconductors and Physics, North University of China, Taiyuan 030051, China; 3 Academy for Advanced Interdisciplinary Research, North University of China, Taiyuan 030051, China; 4 Sichuan Gas Turbine Establishment, Aero Engine Corporation of China, Chengdu 610500, China; 5 School of Instrument and Electronics, North University of China, Taiyuan 030051, China

**Keywords:** graphene, MEMS acceleration sensor, high-g value, high sensitivity, structural optimization, cross-axis sensitivity

## Abstract

High-g accelerometers are in high demand across sectors such as aerospace, defense, and industrial inspection. This paper presents a MEMS accelerometer based on graphene piezoresistors, designed for precise acceleration measurement under sudden impacts, intense vibrations, and extreme conditions, such as engine fault diagnosis and weapon impact testing. A step-by-step structural optimization and simulation analysis were conducted using finite-element simulation. Taking the peak strain at the beam root, the first-order natural frequency, and the maximum equivalent stress as optimization objectives, progressive parametric optimization was sequentially performed on four progressive architectures: a simple beam, a beam mass, a beam mass with stress concentration grooves, and a beam mass with stress concentration grooves and symmetric masses. The results indicate that the introduction of a central mass enhances the peak strain by more than 15 times compared to the simple beam. The addition of stress concentration grooves further increases the strain by approximately 30%. Finally, the incorporation of symmetric masses yields a further 9% strain enhancement while reducing cross-axis sensitivity by 5.6%, effectively suppressing off-axis interference. The final structure achieves maximized strain while maintaining a first-order natural frequency above 200 kHz, with the maximum equivalent stress staying within the allowable limit. This optimal comprehensive performance provides essential technical support for high-performance graphene-based accelerometers. In addition to the mechanical structural optimization, the graphene piezoresistors were treated as surface sensing regions at the beam-root locations, and the area-averaged longitudinal strain was extracted as the input of a piezoresistive transduction model. The simulated strain was converted to resistance variation and bridge output voltage using a graphene gauge-factor-based readout model incorporating contact-resistance effects, thereby providing a sensor-level electromechanical performance estimation for the proposed high-g accelerometer.

## 1. Introduction

The rapid advancement of MEMS technology has paved new avenues for the miniaturization and integration of vibration sensors. Silicon-based piezoresistive MEMS accelerometers have been extensively employed in fields such as aerospace inertial navigation and structural health monitoring, offering advantages including compact size, low power consumption, and ease of mass production [1,2,3,4]. Nevertheless, the operational stability of conventional silicon-based MEMS accelerometers deteriorates significantly in high-temperature environments, making them ill-suited for the rigorous demands of aero-engine hot-end component monitoring [5]. Wung et al. [6] reported a normalized sensitivity of 3.0015 μV/(*V_exc_*·g) in quasi-static centrifugal tests up to approximately 3000 g while significantly suppressing cross-axis sensitivity. However, its dynamic frequency-response parameters remain largely undisclosed, and its performance under high-frequency vibration requires further validation. Bae et al. [7] developed a silicon accelerometer featuring a suspended piezoresistive bridge for a 2000 g range. This design exhibited a sensitivity of 25.5 μV/g with a nonlinearity error of only 0.2% and survived 4667 g impact testing. Despite its robust overload resistance, the lack of temperature compensation leads to significant zero-bias instability in high-temperature environments, as the piezoresistive coefficient is highly temperature dependent. Link et al. [8] developed a monolithic piezoresistive accelerometer for safety testing with a 2000 g full scale and a wide operating temperature range, although its range remains limited to moderate impact levels. Dong et al. [9] reported an axial-beam piezoresistive accelerometer with a 2000 g range, achieving a sensitivity of 0.11 mV/g at 5 V and a resonance frequency of 31 kHz. Although significant progress has been made in high-g MEMS accelerometers for engine monitoring, existing designs still face challenges such as the trade-off between sensitivity and bandwidth, insufficient suppression of cross-axis coupling, and high structural complexity at higher measurement ranges. In addition to silicon-based piezoresistive MEMS accelerometers, other high-g inertial sensing approaches have also been investigated. Surface acoustic wave accelerometers are particularly attractive for harsh shock environments because they can employ compact, robust, and even passive wireless sensing configurations. Their high shock resistance makes them an important reference for high-g inertial sensing. Compared with SAW accelerometers, the present work focuses on a graphene piezoresistive MEMS architecture, aiming to improve the trade-off among local strain sensitivity, bandwidth, and mechanical reliability within a micromachined beam-mass structure.

In pursuit of high-performance MEMS accelerometers, exploring novel materials and architectures has become imperative. Graphene possesses exceptional electrical and mechanical properties, including a Young’s modulus of approximately 1 TPa [10,11], high stretchability [11], and ultra-high carrier mobility [12], making it a promising material for piezoresistive sensing [13,14,15,16]. Graphene has also been integrated into MEMS devices such as pressure sensors, temperature sensors, and microphones [13,17,18,19]. Existing graphene-based accelerometers have mainly focused on low-g vibration detection or highly sensitive NEMS operation [20,21,22,23], while their applicability to ultra-high-g environments remains insufficiently established. For instance, Maharjan et al. [21] developed an accelerometer using graphene/PVDF nanocomposites for low-frequency vibration, yet its range is limited to 8 g. Ding et al. [22,23] reported NEMS accelerometers featuring suspended graphene membranes and atomically thin structures. While these devices exhibit ultra-high sensitivity, their large suspended areas and insufficient structural stiffness lead to catastrophic failure under high-impact loads, typically restricting their range to below 10 g. Consequently, designing a structure that leverages graphene’s material advantages while balancing natural frequency and mechanical strength is of significant research importance for precise measurement in high-g environments.

Recent work on silicon nanowire array sensors also demonstrates that advanced silicon-based nanostructures can act as highly sensitive, MEMS-compatible transduction channels [24]. Therefore, the present design does not simply compare graphene with bulk silicon. Instead, it positions graphene as one candidate in a broader class of nanostructured sensing materials. The specific advantage pursued here is that graphene can be transferred and patterned as an atomically thin surface piezoresistor on the high-strain beam-root region, thereby separating the silicon load-bearing function from the strain-to-resistance conversion function with negligible influence on the global stiffness and natural frequency.

In this work, a graphene piezoresistive accelerometer designed for a 250,000 g range is proposed. Both mechanical and electrical response models were established. Through progressive parametric optimization of various geometries, the stress, strain, deflection, and natural frequencies were systematically analyzed across different parameters. Ultimately, an innovative sensor architecture was developed. To validate the advantages of the proposed method, the design was compared with existing state-of-the-art sensors. Simulation results indicate the potential advantages of the proposed design, while fabrication and experimental calibration will be carried out in future work.

In the proposed device, the silicon load-bearing structure and the graphene piezoresistive sensing layer serve distinct but complementary functions. The silicon structure governs the global stiffness, natural frequency, deformation behavior, and mechanical strength of the accelerometer, while the ultra-thin graphene piezoresistors located at the beam roots convert the local strain into resistance changes and a corresponding bridge output voltage.

## 2. Working Principle

**Figure 1:**
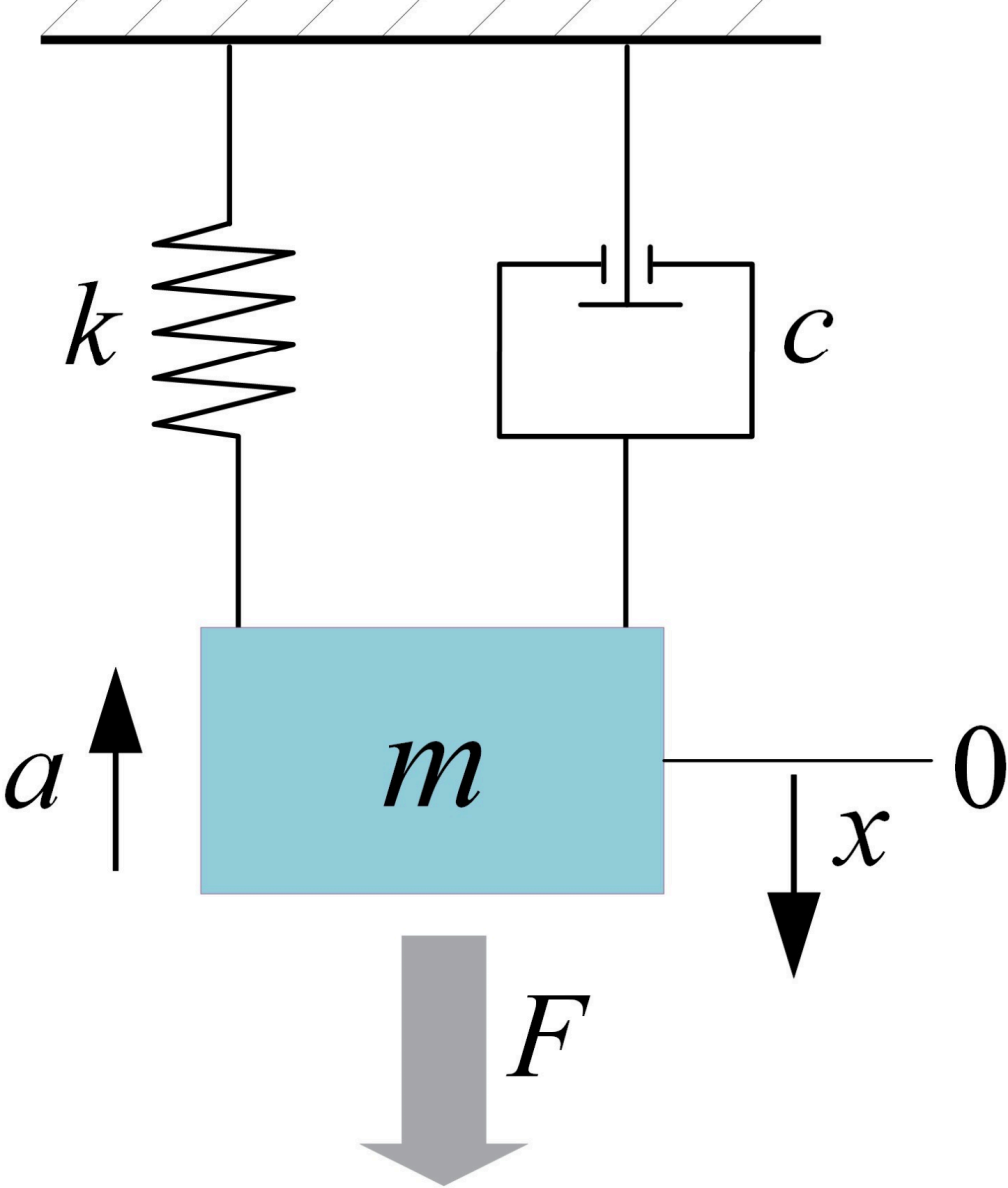
Mass–spring–damper system.

The sensing mechanism of the piezoresistive accelerometer is illustrated in Figure 1. This structure can be modeled as a single-degree-of-freedom second-order mass–spring–damper system, primarily consisting of a proof mass (*m*), structural stiffness (*k*), and an air damping coefficient (*c*).

When the sensor is subjected to an external acceleration *a*, the proof mass experiences an inertial force in the opposite direction: *F* = −*ma*. This inertial force induces a relative displacement of the mass *x*(*t*), which in turn causes bending deformation of the sensing beams [25]. The governing equation of motion for the system is determined by the coupling of Newton’s second law and Hooke’s law:

(1) $$m\frac{d^{2}x\left(t\right)}{dt^{2}}+c\frac{dx\left(t\right)}{dt}+kx\left(t\right)=F\left(t\right)$$

In this design, four graphene piezoresistors are precisely positioned at the fixed root of the sensing beams. Finite-element analysis indicates that this region exhibits the maximum surface stress distribution under inertial loading. These four graphene piezoresistors function as four independent active bridge arms, each of which can be integrated with three external fixed-arm resistors within an external signal conditioning module. This configuration not only simplifies the routing process but also enhances the flexibility of the testing system. The output signal of the bridge directly reflects the relationship between the input excitation voltage *V_s_* and the fractional change in the resistance of the bridge arms. For the quarter-bridge configuration with one active graphene piezoresistor, the theoretical output-voltage relationship is:

(2) $$\frac{V_{out}}{V_{s}}=\frac{\Delta R}{4R_{0}+2\Delta R}$$

where *R*_0_ denotes the initial resistance of the graphene piezoresistor without external excitation and Δ*R* represents the dynamic resistance increment relative to acceleration changes. Based on Equation (2), the primary objective of this design is to achieve high sensitivity while simultaneously ensuring high-frequency response and mechanical reliability.

In the finite-element model, graphene was not explicitly meshed as a three-dimensional load-bearing solid because the atomic-scale thickness of monolayer graphene is several orders of magnitude smaller than the silicon sensing beams. Instead, graphene was represented by predefined surface piezoresistive sensing regions located at the beam roots. The area-averaged longitudinal strain extracted from these regions was used as the input of the graphene piezoresistive model. The graphene-specific parameters used in the sensor-level estimation include the gauge factor (GF), intrinsic graphene resistance (*R_g_*), contact resistance (*R_c_*), and bridge excitation voltage (*V_s_*); the monolayer thickness and elastic modulus are used only to justify why graphene is not treated as the dominant load-bearing layer.

For a practical graphene piezoresistor, the initial intrinsic resistance can be estimated from the sheet resistance and geometry as:

(3) $$R_{g}=\frac{R_{sh}\cdot L_{g}}{W_{g}}$$

where *R_sh_* is the graphene sheet resistance, *L_g_* is the patterned graphene length, and *W_g_* is the graphene width. The key process-dependent parameters that should be characterized experimentally include Raman indicators of graphene quality, sheet resistance, gauge factor, transfer-induced cracks/wrinkles, and metal–graphene contact resistance obtained by four-probe or transmission-line-method measurements. Because these parameters depend strongly on the graphene growth, transfer, patterning, and contact process, the present simulation uses representative GF values and a contact-resistance correction to provide a transparent sensor-level estimation rather than claiming a fixed experimental electrical output:

(4) $$\frac{\Delta R}{R_{0}}=GF\varepsilon_{l}$$

where *R*_0_ is the initial intrinsic resistance of the graphene strip, Δ*R* is the resistance change, and ε*_l_* is the area-averaged longitudinal strain along the graphene strip. Here, *R*_0_ is equivalent to *R_g_* in Equation (3), while the metal–graphene contact resistance is introduced separately in Equation (6).

To convert the graphene resistance change described by Equation (4) into a measurable electrical signal, each graphene piezoresistor is evaluated using a quarter-bridge circuit, in which the graphene element serves as the single active arm and the remaining three arms are fixed resistors. Accordingly, the calculated voltage represents the output of one graphene piezoresistor located in the high-strain region at the beam root. Assuming that Δ*R* ≪ *R*_0_, the bridge output voltage can be approximated as:

(5) $$V_{out}\approx \left(\frac{V_{s}}{4}\right)\left(\frac{\Delta R}{R_{0}}\right)=\frac{V_{s}GF\varepsilon_{l}}{4}$$

where *V_s_* is the bridge excitation voltage. Because graphene contact resistance depends strongly on transfer quality and metal contact formation, the effective resistance variation was also evaluated using:

(6) $${\left(\frac{\Delta R}{R_{0}}\right)}_{eff}=\frac{R_{g}}{R_{g}+2R_{c}}GF\varepsilon_{l}$$

where *R_g_* is the intrinsic resistance of the graphene strip and *R_c_* is the contact resistance at each metal–graphene interface.

To further clarify the material origin of the gauge factor in Equation (4), the resistance response of graphene under small uniaxial strain is related to the strain-induced variation in Fermi velocity. Based on the strain-dependent Fermi-velocity model in Ref. [26] and the adopted carrier-transport approximation, the gauge factor can be written as:

(7) $$GF=\frac{\Delta R/R_{0}}{\varepsilon_{l}}=1+\nu -2\frac{\Delta v_{F}}{v_{F}\varepsilon_{l}}$$

where ν is the effective transverse contraction ratio of the graphene–support system, v_F_ is the initial Fermi velocity, Δv_F_ is the strain-induced variation in Fermi velocity, and ε_l_ is the area-averaged longitudinal strain. Based on the anisotropic Fermi-velocity expressions of graphene under uniaxial strain reported by Oliva-Leyva and Naumis [26], the following idealized limiting gauge factors can be derived for the armchair and zigzag directions:

(8) $${\mathrm{GF}}_{\mathrm{arm}}\;=\;1\;+\;\nu \;-\;2\left(1\;-\;\beta \right)$$

(9) $${\mathrm{GF}}_{\mathrm{zig}}=\;1+\nu +2\nu \left(1\;-\;\beta \right)$$

where β is the hopping Grüneisen parameter of graphene. Equations (8) and (9) represent idealized crystallographic limiting cases under uniform uniaxial strain rather than experimentally calibrated gauge factors of the present device.

By substituting the gauge factor into the quarter-bridge model in Equation (5) and applying the contact-resistance attenuation defined in Equation (6), the effective bridge output can be directly related to the graphene material response, contact resistance, and FEA-extracted longitudinal strain:

(10) $$V_{out,eff}=\frac{V_{s}}{4}\frac{R_{g}}{R_{g}+2R_{c}}GF\varepsilon_{l}$$

Accordingly, the idealized output-voltage expressions for the armchair and zigzag limiting directions are, respectively:

(11) $$V_{out,arm}=\frac{V_{s}}{4}\frac{R_{g}}{R_{g}+2R_{c}}\left[1+\nu -2\left(1-\beta \right)\right]\varepsilon_{l}$$

(12) $$V_{out,zig}=\frac{V_{s}}{4}\frac{R_{g}}{R_{g}+2R_{c}}\left[1+\nu +2\nu \left(1-\beta \right)\right]\varepsilon_{l}$$

Equations (10)–(12) are valid under the small-resistance-change condition Δ*R* ≪ *R_0_* and assume that the metal–graphene contact resistance is independent of strain. Because the crystallographic orientation and process-dependent gauge factor of the transferred graphene have not been experimentally calibrated for the proposed device, representative gauge factors are used for parametric evaluation. Previous experiments reported an average gauge factor of 2.92 and a maximum value of 4.33 for suspended graphene membranes [27], while a substantially higher gauge factor of approximately 150 was measured for mechanically exfoliated graphene piezoresistors fabricated on silicon wafers [28]. Therefore, GF = 2, 4, and 10 were selected as the baseline, intermediate, and upper parametric cases, respectively. GF = 10 is used only as an upper parametric case and is not regarded as an experimentally calibrated value of the proposed accelerometer. *V_s_* was set to 5 V for the sensor-level output estimation.

Equations (4)–(12) establish a sequential electromechanical relationship linking the FEA-extracted graphene strain, the material-dependent gauge factor, resistance variation, metal–graphene contact-resistance attenuation, and bridge output voltage, while the silicon structure remains the dominant mechanical load-bearing layer.

The effective operating bandwidth of the sensor is limited by its first-order natural frequency. Although reducing the natural frequency can significantly enhance mechanical sensitivity, it severely compresses the measurable frequency range of the device. Furthermore, dynamic stability is a critical factor in the design process: when the external vibration frequency approaches the system’s natural frequency, resonance will be triggered. This not only causes nonlinear distortion of the output signal but the excessive amplitude may also lead to fatigue damage or catastrophic fracture of the sensing beams. To ensure structural integrity and signal fidelity, the first-order natural frequency must be designed beyond the upper limit of the target frequency band. Its value is co-determined by the structural stiffness k and the mass of the proof mass m:

(13) $$f_{0}=\frac{1}{2\pi }\sqrt{\frac{k}{m}}$$

According to the amplitude–frequency characteristics of the sensor, the upper limit of the working frequency is typically set at one-third of the first-order natural frequency to minimize amplitude errors [29]. That is:

(14) $$f_{0}>3f_{max}$$

To ensure the linearity of the sensor, the structural deformation must adhere to the small-deflection theory, which stipulates that the maximum deflection *w_max_* should not exceed one-fifth of the thickness at the thinnest part of the structure *h_min_*. When the inertial force is applied to the proof mass, it induces deformation in the sensing beams. Analogous to the small-deflection theory for diaphragms, the deformation of the sensing beams must satisfy [30]:

(15) $$w_{max}\ll \frac{h_{min}}{5}$$

Although Ref. [30] reports a pressure sensor rather than an accelerometer, it is cited here only for the small-deflection design criterion and not as a direct accelerometer precedent. The physical basis is the same continuum-mechanics requirement: the deformation of the strain-sensitive structure should remain small compared with its characteristic thickness so that the strain field and the piezoresistive response remain approximately linear. In the present accelerometer, the external acceleration is converted into inertial force on the proof mass, and the resulting beam bending provides the local strain that is read by the graphene piezoresistor.

To ensure structural reliability, the maximum equivalent stress must satisfy the following safety criterion [31]:

(16) $$\sigma_{\max}\leq [\sigma ]=\frac{\sigma_{s}}{n}$$

where [σ] represents the allowable stress. In this study, the safety factor *n* is set to 10, and σ*_s_* is taken as 7 GPa [32]. Furthermore, to ensure high sensitivity, the strain ε at the root of the sensing beams is targeted to exceed approximately 3000 με. Although graphene has an extremely high Young’s modulus and intrinsic strength, the graphene piezoresistive layer is atomically thin compared with the silicon sensing beams with thicknesses of tens of micrometers. Therefore, its contribution to the global bending stiffness, natural frequency, and load-bearing strength of the structure is neglected in the present mechanical model. The strength criterion in Equation (16) is thus applied to the silicon structural layer, while graphene is treated primarily as the piezoresistive sensing layer.

## 3. Structural Design and Optimization

A novel accelerometer architecture was designed in this work and simulated using the anisotropic material properties of silicon. This structure is capable of measuring high-g accelerations while maximizing sensitivity under the prerequisite of a high-frequency response. The objective of this study is to achieve a global optimization of the local strain for graphene piezoresistors while balancing structural miniaturization with strain uniformity.

The structural response was calculated using a three-dimensional finite-element model. Because the device layer is single-crystal silicon, anisotropic elastic properties were adopted rather than an isotropic approximation. The stiffness matrix was defined according to the crystallographic orientation of silicon using the elastic constants *C*_11_, *C*_12_, and *C*_44_ from the literature and material-database values. Fixed constraints were applied at the outer anchors, and acceleration loading corresponding to the target high-g input was imposed on the whole structure. Mesh refinement was applied near the beam roots and stress concentration grooves, where large strain and stress gradients occur. Mesh convergence was checked by comparing the area-averaged strain in the graphene sensing region, maximum deflection, and maximum equivalent stress under successively refined mesh sizes. The present finite-element simulations were performed under room-temperature and isothermal conditions, and thermal stress was not included in the model. Therefore, the reported mechanical and electrical performance does not account for thermally induced deformation or initial pre-strain.

**Figure 2:**
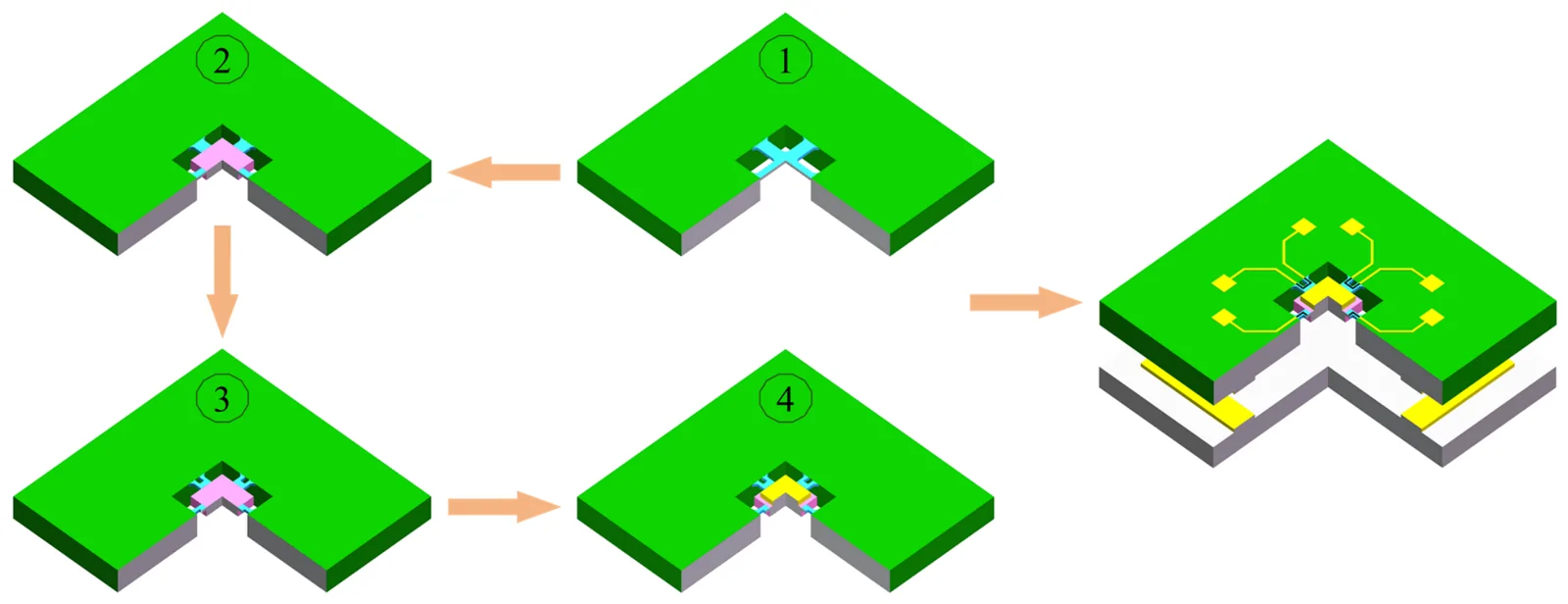
Acceleration sensor structure optimization process.

Figure 2 illustrates the proposed four-stage progressive structural optimization path. Structure ① is a basic cross-beam, serving as the design baseline. Structure ② incorporates a central proof mass to amplify inertial forces and augment strain output. Structure ③ introduces stress concentration grooves (SCGs) at the beam-mass junctions to further increase the peak strain at the beam roots via local cross-sectional reduction. Finally, Structure ④ adds symmetric masses atop the central mass to mitigate inertial coupling and reduce cross-axis sensitivity. Through this sequential optimization, the complete sensor chip structure shown on the right was obtained.

The optimization procedure in this study is a progressive parametric optimization rather than a free-form topology optimization. The cross-beam structure first defines the global beam dimensions under frequency and stress constraints. The central proof mass is then introduced to increase inertial force. Stress concentration grooves are subsequently used as local strain-regulation features after the global beam dimensions and mass size have been constrained. Finally, symmetric masses are added to suppress cross-axis coupling. Thus, each step provides a constraint or baseline for the next step rather than replacing the previous design decision.

### 3.1. Design of Cross-Beam Structure

As a common sensing configuration, the geometric dimensions of the cross-beam directly dictate key performance metrics, such as sensitivity, linearity, and frequency response. In this section, the cross-beam is first utilized as the baseline structure to optimize the beam length, width, and thickness, thereby establishing a design foundation for the subsequent integration of the proof mass and localized stress regulation features.

**Figure 3:**
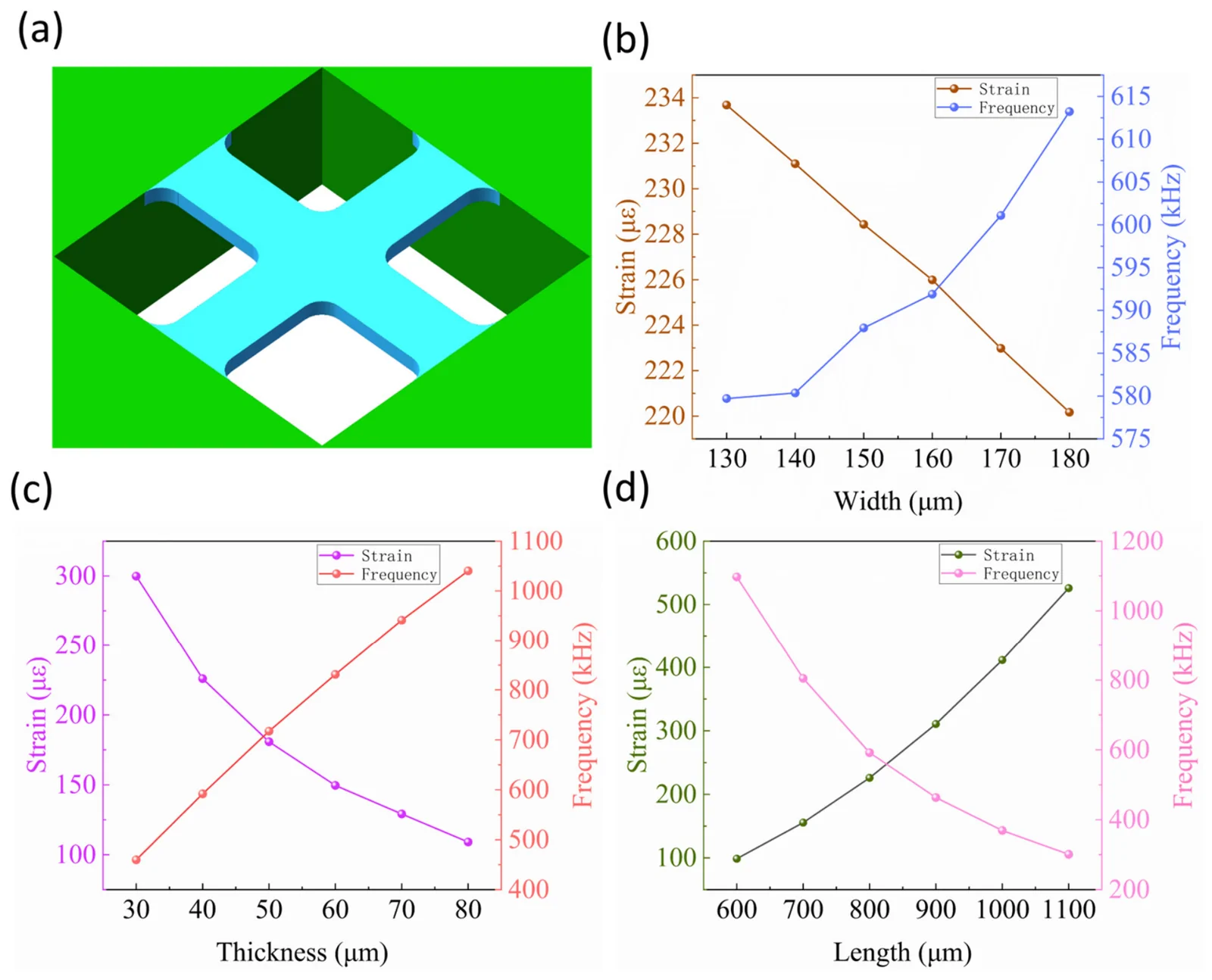
(**a**) Cross-beam structure. (**b**) Effect of beam width on strain and natural frequency. (**c**) Effect of beam thickness on strain and natural frequency. (**d**) Effect of beam length on strain and natural frequency.

As illustrated in Figure 3a, fillets are incorporated at the intersections of the cross-beam to prevent stress concentration. Simultaneously, the local transition radius at the junction nodes is enlarged to improve the uniformity of the stress distribution under inertial loading.

Figure 3b–d illustrate the combined effects of beam width, thickness, and length on the strain and natural frequency. The results indicate that an increase in beam width enhances structural stiffness, leading to a synchronous rise in the first-order natural frequency, while the strain exhibits an overall downward trend. Specifically, as the beam width increases from 130 μm to 180 μm, the strain at the beam root gradually decreases from approximately 234 με to 220 με, while the natural frequency improves from 579 kHz to 613 kHz. To maintain sufficient strain output while avoiding fabrication complexities and structural integrity risks associated with excessively narrow beams, a width of 160 μm was selected.

The influence of beam thickness and length is even more pronounced. As the thickness increases from 30 μm to 40 μm, the structural stiffness rises rapidly, resulting in a 26% increase in natural frequency but a 24% reduction in root strain. A further increase to 50 μm leads to an additional 19.7% drop in strain, which is detrimental to the piezoresistive signal output. Regarding beam length, an increase from 600 μm to 1100 μm enhances the strain by 47% but reduces the natural frequency by 43%. Notably, beyond a length of 800 μm, the frequency decline accelerates while the strain gain becomes relatively limited. Balancing sensitivity, frequency response, and structural reliability, the optimal dimensions for the cross-beam were determined to be a length of 800 μm, a width of 160 μm, and a thickness of 40 μm, providing a baseline for the subsequent proof mass design.

### 3.2. Beam-Mass Structure Design

**Figure 4:**
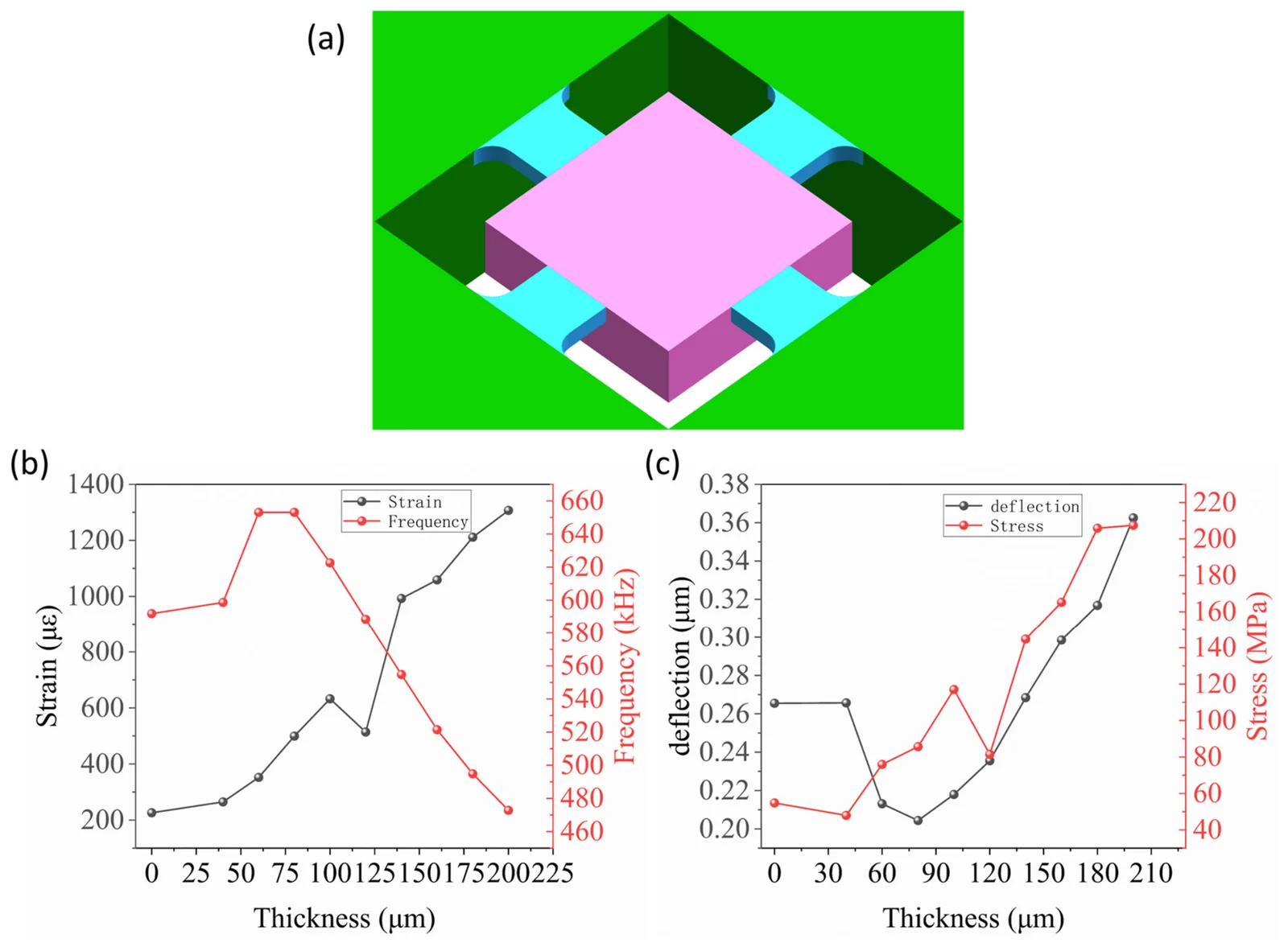
(**a**) Cross-beam with mass block structure. (**b**) Effect of mass block thickness on strain and natural frequency. (**c**) Effect of mass block thickness on deflection and stress.

Based on the optimal dimensions of the cross-beam, a central proof mass is introduced to increase the inertial force, thereby enhancing the sensor sensitivity. As illustrated in Figure 4a, the proof mass is located at the center of the cross-beam and is monolithically connected to the four cantilever beams. Given that thickness exerts the most significant influence on sensor performance, the length and width of the mass are fixed at 500 μm. Parametric optimization of the mass thickness was conducted within a range of 60 to 200 μm with a step size of 20 μm.

Figure 4b,c illustrate the influence of proof mass thickness on structural performance. As the thickness increases, the effective mass and the corresponding inertial force rise synchronously, leading to a significant enhancement in beam-root strain and overall sensitivity. However, this increase in system mass simultaneously causes a decline in the first-order natural frequency. These findings indicate that while increasing the mass thickness benefits sensitivity, it compromises the high-frequency response capability of the structure.

In accordance with Equation (13) and the bandwidth constraint in Equation (14), the trade-off between strain and natural frequency was comprehensively analyzed. When the mass thickness is small, the strain enhancement at the beam root is limited. Although further thickening amplifies the strain output, it accelerates the decline in frequency, which is unfavorable for meeting the wide-bandwidth requirements of high-g sensors. Balancing the magnitude of sensitivity improvement, natural frequency, and structural stability, the proof mass thickness was finalized at 140 μm. This resulted in an optimized beam-mass configuration, providing a foundation for subsequent local stress regulation.

### 3.3. Beam Mass with Stress Concentration Groove Design

**Figure 5:**
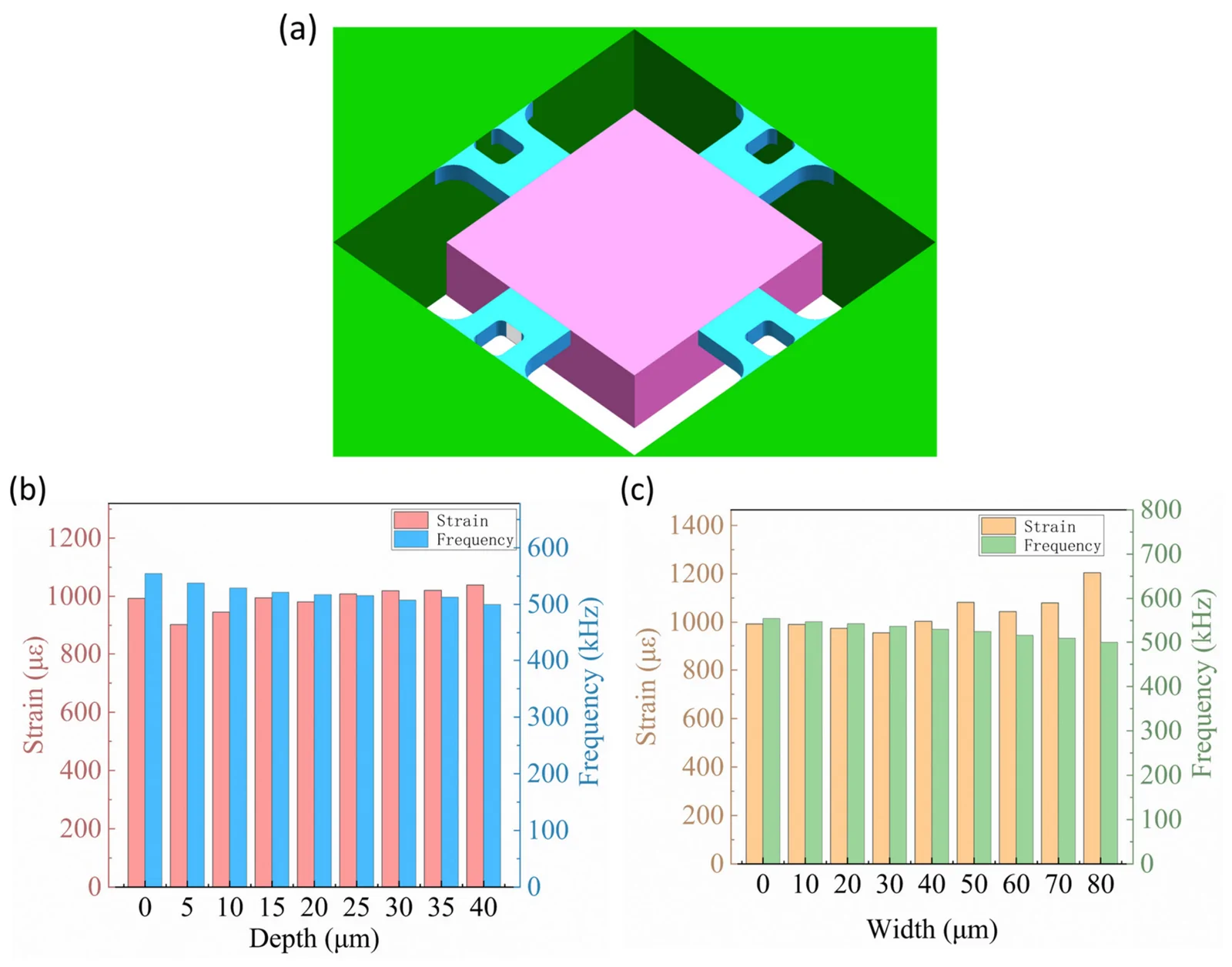

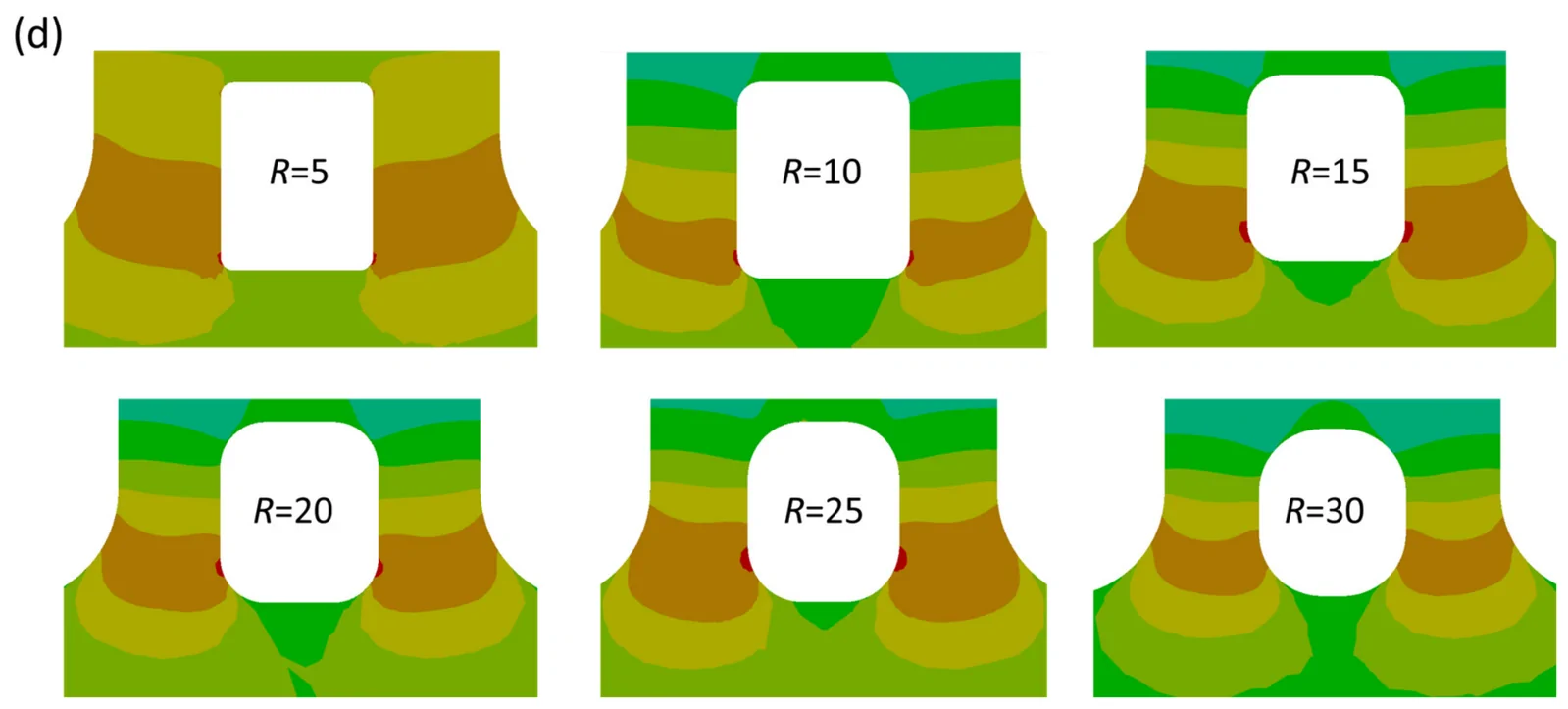
(**a**) Structure with stress concentration grooves. (**b**) Effect of groove depth on performance. (**c**) Effect of groove width on performance. (**d**) Effect of fillet on stress concentration.

To further enhance the localized strain at the beam roots, stress concentration grooves were incorporated on both sides of the junctions between the cross-beams and the proof mass. As illustrated in Figure 5, the SCGs are positioned at the fixed ends of the beams to amplify local strain by reducing the effective cross-sectional area. Simultaneously, fillets were applied at the ends of the grooves to attenuate secondary stress concentrations and prevent fatigue fracture. With the groove length fixed at 80 μm, three parameters—groove width (30–100 μm), depth (20–60 μm), and fillet radius (5–25 μm)—were sequentially optimized.

Figure 5b illustrates the influence of groove depth: as the depth increases from 0 μm to 40 μm, the strain increases by 6.3% at the expense of a 9.6% decline in the natural frequency. The impact of groove width on the beam-root strain is shown in Figure 5c. With the expansion of the groove width, the local cross-section is weakened, resulting in an overall upward trend in strain. Specifically, increasing the width from 0 μm to 70 μm yields an 8.5% enhancement in peak strain, while the first-order natural frequency decreases by 8%. The optimization results for the fillet radius, presented in Figure 5d, demonstrate that increasing the radius from 5 μm to 15 μm significantly contracts the high-stress concentration zone, indicating that the stress at sharp corners is effectively alleviated. However, further increasing the radius weakens the stress concentration effect, leading to a diminished strain gain.

Based on the optimization of these three parameters, the final optimal geometric parameters for the SCGs were determined as a width of 70 μm, a depth of 40 μm, and a fillet radius of 15 μm. Under this configuration, the peak strain at the beam root is significantly enhanced compared to the groove-less structure. Meanwhile, the maximum equivalent stress remains within the allowable limits, and the first-order natural frequency stays within the target range, achieving optimal comprehensive structural performance.

After the introduction of SCGs, the graphene piezoresistors are placed on the top surface of the sensing beams near the fixed beam-root regions, adjacent to the groove-root transition where the longitudinal surface strain is maximized. The resistors are not arranged on the groove sidewalls, which avoids discontinuities, sharp corners, and potential fabrication defects while still capturing the amplified tensile/compressive strain.

The use of local stress concentration is related to earlier work in which slots were etched into the flexures of a silicon piezoresistive micro-accelerometer to improve sensitivity [33]. However, the present structure differs in both device objective and sensing implementation. First, the SCGs are optimized together with a bottom proof mass and a symmetric top mass for an ultra-high target range of 250,000 g and a first-order natural frequency above 200 kHz, whereas the earlier slotted-flexure accelerometer addressed a much lower-frequency micro-accelerometer. Second, the present SCGs are used to define high-strain surface regions for graphene piezoresistors, and the sensor response is evaluated from area-averaged graphene strain and bridge output estimation rather than from boron-diffused silicon piezoresistors. Thus, the groove design is not a direct duplication of the previous slot structure but a geometry adapted to the proposed graphene surface readout and high-g design constraints.

### 3.4. Symmetrical Proof Mass Design

**Figure 6:**
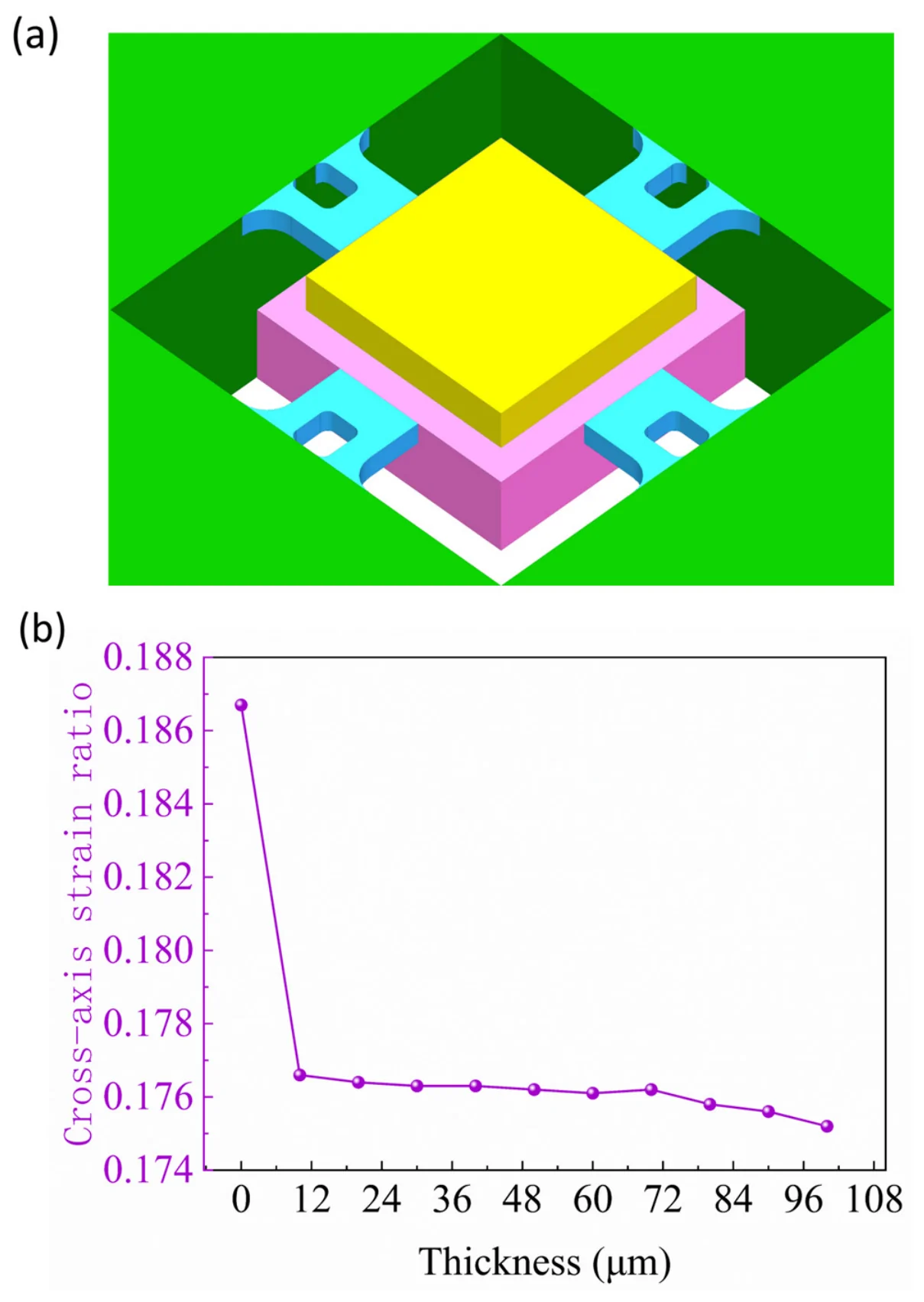
(**a**) Symmetric mass block structure. (**b**) Effect on cross-axis sensitivity.

Traditional cross-beam structures with a single proof mass inherently exhibit significant cross-axis output when subjected to lateral acceleration, primarily due to structural asymmetry. This leads to increased measurement errors for single-axis sensors. To address this issue, this section proposes a symmetric mass architecture. As illustrated in Figure 6a, an Au symmetric top mass is added above the central silicon proof mass. In the finite-element model, Au was treated as an isotropic material with a Young’s modulus of 78 GPa, a Poisson’s ratio of 0.44, and a density of 19,300 kg/m^3^. These material properties were used in the geometric optimization, stress analysis, and modal analysis. This configuration balances the mass distribution along the transverse axis, thereby suppressing inertial coupling in the cross-axis directions.

Figure 6b presents a comparison of the output responses between structures with and without the symmetric mass under cross-axis acceleration. The results indicate that as the thickness of the symmetric mass increases from 0 μm to 10 μm, the cross-axis sensitivity decreases by 5.4%, demonstrating that even a small thickness range can significantly improve lateral inertial coupling. As the thickness continues to increase, the rate of decline gradually levels off. In the finalized design, the cross-axis sensitivity is cumulatively reduced by 5.6% compared to the structure without a symmetric mass, effectively suppressing the cross-talk of lateral acceleration on the primary axis signal.

**Figure 7:**
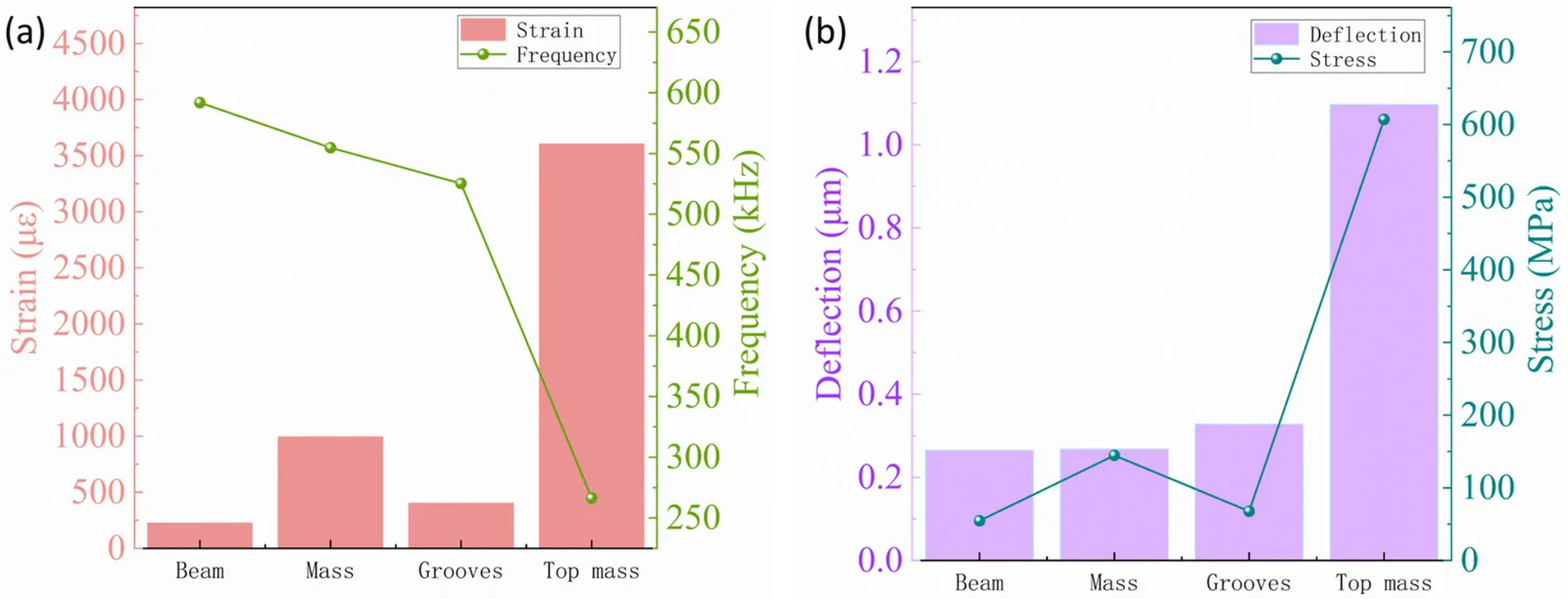
(**a**) Effect of four structures on strain and natural frequency. (**b**) Effect of four structures on deflection and stress.

Figure 7 provides a comprehensive performance comparison of the four optimized structures. Compared with the basic cross-beam, the beam-mass structure, and the beam-mass–SCG structure, the introduction of symmetric masses maintains structural deflection and stress within acceptable limits while achieving the highest strain levels among the four configurations, which is conducive to enhancing piezoresistive output. Although the first-order natural frequency decreases further, it remains above 200 kHz, satisfying the requirements for high-frequency response. Consequently, the symmetric mass structure achieves an optimal trade-off between sensitivity and dynamic performance while improving cross-axis sensitivity, and is thus finalized as the proposed design for this work.

From an implementation viewpoint, the symmetric proof mass is designed as a comparatively large-area mass feature located on the central proof mass rather than as a fragile nanoscale suspended element. It can therefore be realized by controlled electroplating, metal bonding, or wafer-level bonding after the silicon beam-mass structure is formed. The main reliability risks are mass-thickness nonuniformity, alignment error, residual stress, and interface delamination under high-g shock loading. These risks can be mitigated by using filleted transitions, symmetric layout alignment marks, low-stress deposition or bonding processes, and post-fabrication modal/shock calibration. The simulated stress distribution indicates that the optimized structure remains within the silicon allowable-stress criterion, providing the mechanical basis for subsequent fabrication verification.

For the final structure, the first-order natural frequency remains above 200 kHz. According to the common design criterion that the usable bandwidth should be lower than approximately one-third of the first-order natural frequency to reduce dynamic amplitude error, the estimated operating bandwidth is above 66 kHz. Because the gauge factor and contact resistance depend on the specific graphene growth, transfer, and patterning process, the present work reports the simulation-based strain response and sensitivity estimation method rather than an experimentally measured electrical sensitivity.

**Figure 8:**
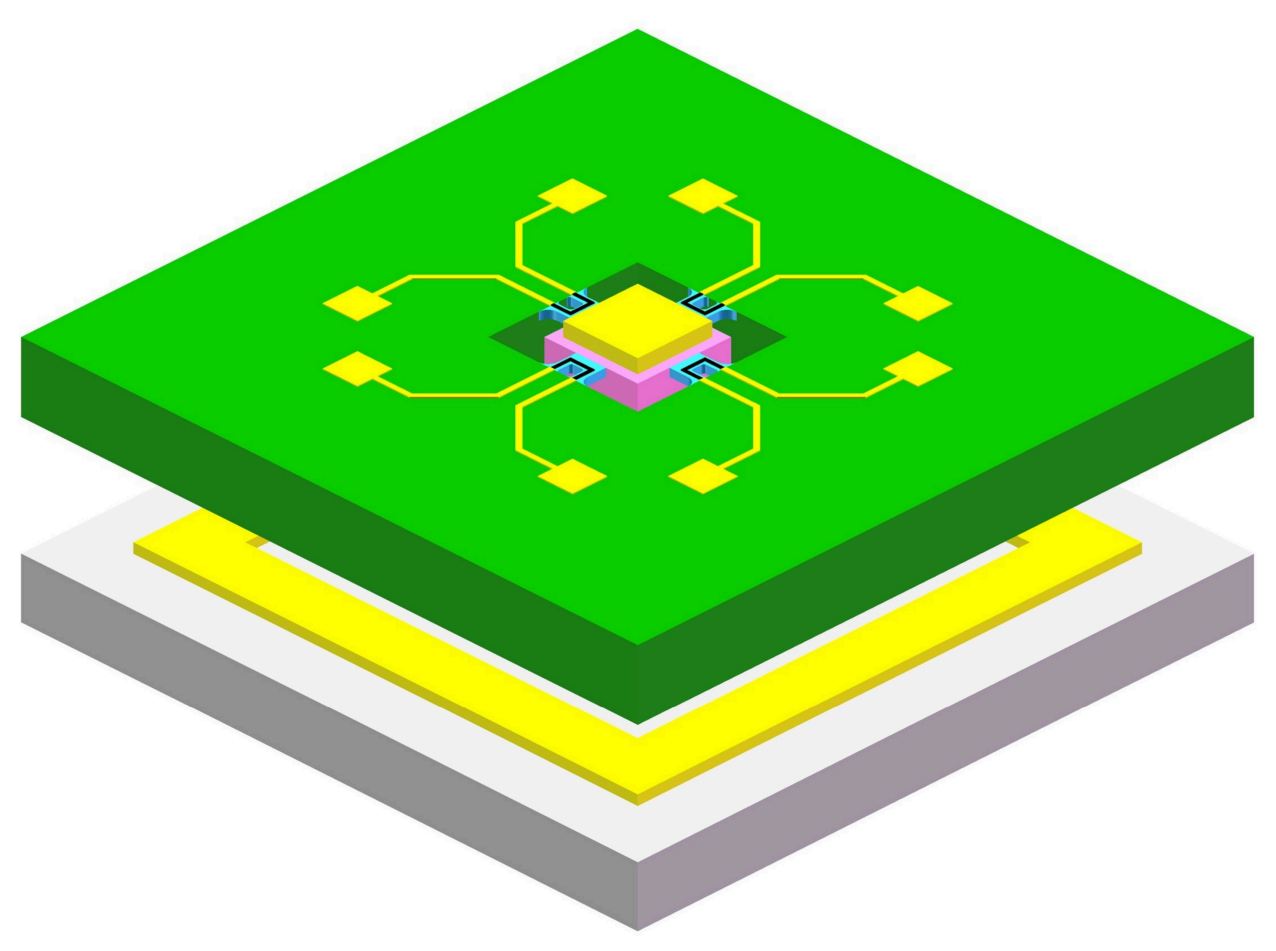
Schematic diagram of an accelerometer chip structure.

Following the step-by-step optimization, the final accelerometer chip architecture is illustrated in Figure 8. The key dimensions of the cross-beam and SCGs are listed in Table 1, while the dimensions of the symmetric mass are provided in Table 2. This structure ensures high localized strain output while balancing structural stiffness and frequency response, establishing a geometric baseline for subsequent device fabrication and experimental validation.

### 3.5. Graphene Piezoresistor Layout and Sensor-Level Performance Estimation

**Figure 9:**
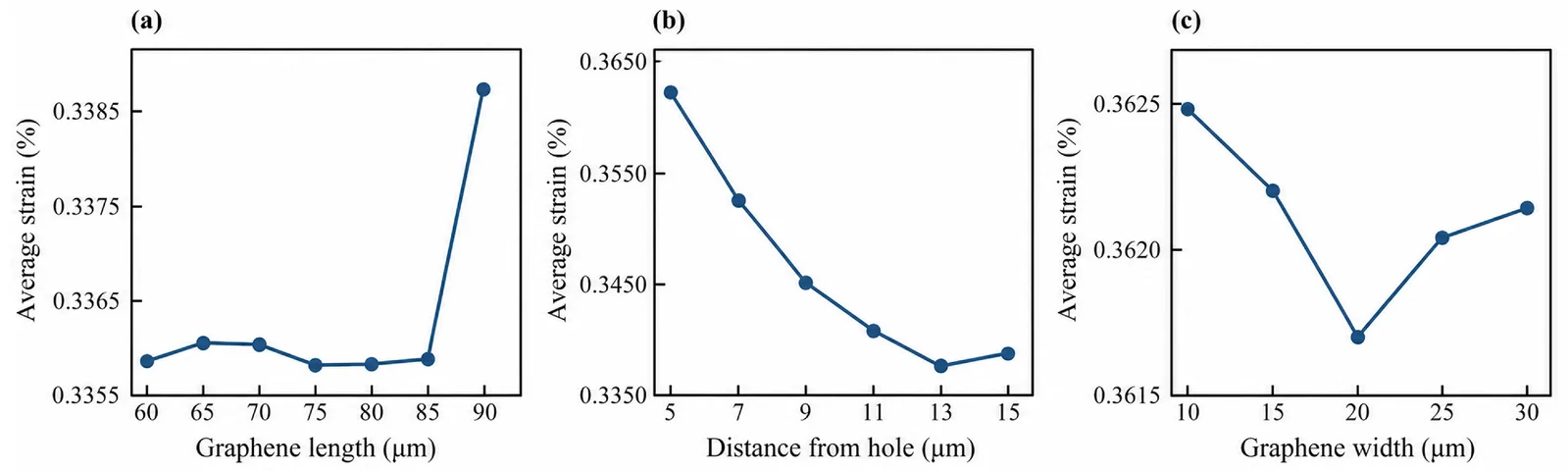
Effects of graphene piezoresistor layout parameters on the area-averaged strain: (**a**) graphene length, (**b**) distance from the hole, and (**c**) graphene width.

To support the graphene-based piezoresistive sensing claim, the geometric layout of the graphene piezoresistor was further evaluated by extracting the area-averaged strain over predefined graphene regions. The effects of graphene length, distance from the hole, and graphene width are shown in Figure 9. The results indicate that the graphene length and width have limited influence on the averaged sensing strain, whereas the distance from the hole is more critical. When the distance decreases from 15 μm to 5 μm, the averaged strain increases from 0.33873% to 0.36220%, corresponding to an increase of approximately 6.48%. Therefore, the graphene piezoresistor should be located close to the high-strain region while maintaining a reasonable fabrication margin.

Using the representative area-averaged longitudinal strain of ε*_l_* = 0.36220% and a bridge excitation voltage of *V_s_* = 5 V, the ideal full-scale quarter-bridge outputs estimated by Equation (5) are 9.06 mV, 18.11 mV, and 45.28 mV for GF = 2, 4, and 10, respectively. However, the metal–graphene contact resistance can attenuate the effective resistance variation and output voltage. Assuming a moderate contact-resistance ratio of *R_c_* = 0.5*R_g_*, the attenuation factor *R_g_*/(*R_g_* + 2*R_c_*) in Equation (6) becomes 0.5. Therefore, the corresponding effective full-scale bridge outputs are estimated to be 4.53 mV, 9.06 mV, and 22.64 mV for GF = 2, 4, and 10, respectively. For a full-scale acceleration of 250,000 g, the effective voltage sensitivities are approximately 18.1 nV/g, 36.2 nV/g, and 90.6 nV/g.

Although the voltage sensitivity is in the nV/g range because of the ultra-high measurement range, the full-scale output remains in the millivolt range and can be further amplified by a low-noise instrumentation amplifier in practical readout circuits. These results indicate that minimizing the metal–graphene contact resistance is critical for preserving the sensor-level output signal. The linearity of the estimated electrical output is mainly governed by the linearity of the structural strain response and the assumed linear piezoresistive relation in Equation (4). Since the maximum deformation of the sensing beam remains within the small-deflection criterion defined in Equation (15), the strain response is expected to remain approximately linear within the investigated full-scale acceleration range. A more rigorous nonlinearity evaluation based on multi-point dynamic calibration will be carried out in future experimental work.

**Figure 10:**
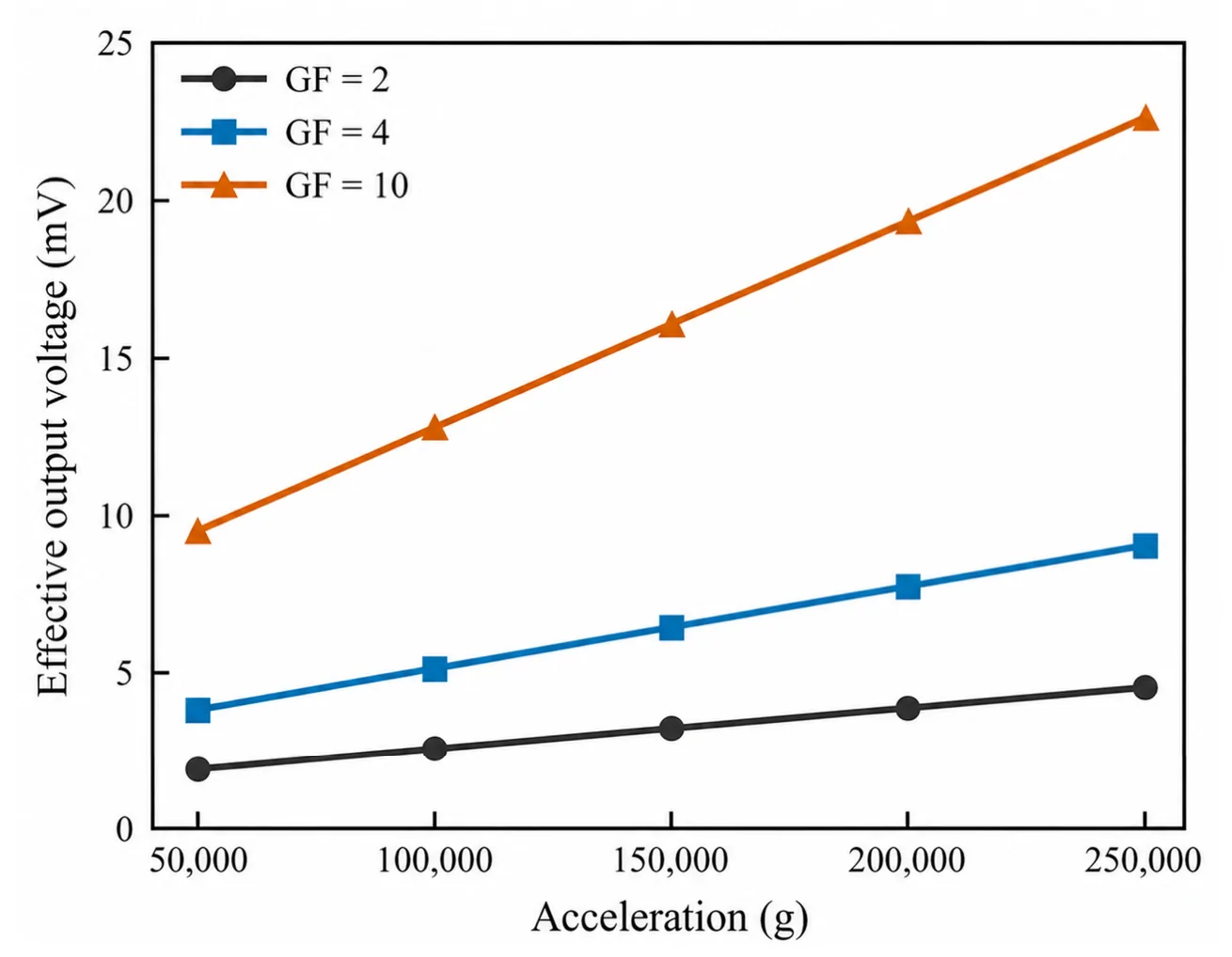
Contact-resistance-corrected bridge output voltage versus acceleration under different graphene gauge factors. The effective output was calculated using the area-averaged graphene sensing strain, a bridge excitation voltage of 5 V, and a moderate contact-resistance ratio of *R_c_* = 0.5*R_g_*.

To further evaluate the acceleration-dependent sensor-level electrical response, multi-point finite-element simulations were conducted at 50,000 g, 100,000 g, 150,000 g, 200,000 g, and 250,000 g. For each acceleration level, the area-averaged longitudinal strain in the graphene sensing region was extracted and converted into the contact-resistance-corrected bridge output voltage using the graphene piezoresistive model. As shown in Figure 10, the effective output voltage increases approximately linearly with acceleration, and a larger graphene gauge factor leads to a stronger electrical response. For *R_c_* = 0.5*R_g_*, the estimated full-scale outputs at 250,000 g are 4.53 mV, 9.06 mV, and 22.64 mV for GF = 2, 4, and 10, respectively. These results further support the feasibility of converting the FEA-extracted graphene-region strain into a measurable bridge output signal.

### 3.6. Functional Role of Graphene and Comparison with Conventional Silicon-Based Piezoresistive Accelerometers

Table 3 quantitatively compares the proposed device with selected silicon- and graphene-based accelerometers reported in the literature [4,5,6,7,9,20,34]. The comparison focuses on measurement range, resonant frequency, estimated output at the stated input, and validation basis. Dagger-marked values are derived from data explicitly reported in the cited literature and the input conditions stated in the corresponding rows. The source bias convention is retained: absolute outputs are reported in the units shown in the table, whereas voltage-normalized outputs are given in mV/V_exc_. The proposed-device output is an analytical estimate based on the FEA-extracted strain.

Table 3 shows that the proposed accelerometer combines a 250,000 g target range with a simulated first-mode resonant frequency above 200 kHz and an analytically estimated bridge output of 4.53–22.64 mV at the target input and 5 V excitation. Within the selected comparison set, and on a simulation/analytical basis, the design combines a high target range and high resonant frequency while retaining a millivolt-level estimated electrical output.

### 3.7. Fabrication Feasibility and Practical Implementation

The front-side graphene and metal-contact processes are completed while the SOI wafer remains mechanically supported and approximately planar. After deposition of the insulating/passivation layer, monolayer CVD graphene is transferred and patterned at the beam-root sensing regions, followed by Cr/Au contact formation and graphene passivation with contact-pad opening. Backside lithography and partial DRIE are subsequently performed while temporary structural support is retained. The Au symmetric top mass is then integrated by electroplating or bonding, and the accelerometer structure is released during the final dry-etching step. This sequence avoids transferring graphene onto a fully released non-planar structure and reduces the risks of graphene cracking, wrinkling, and unstable metal–graphene contact resistance.

**Figure 11:**
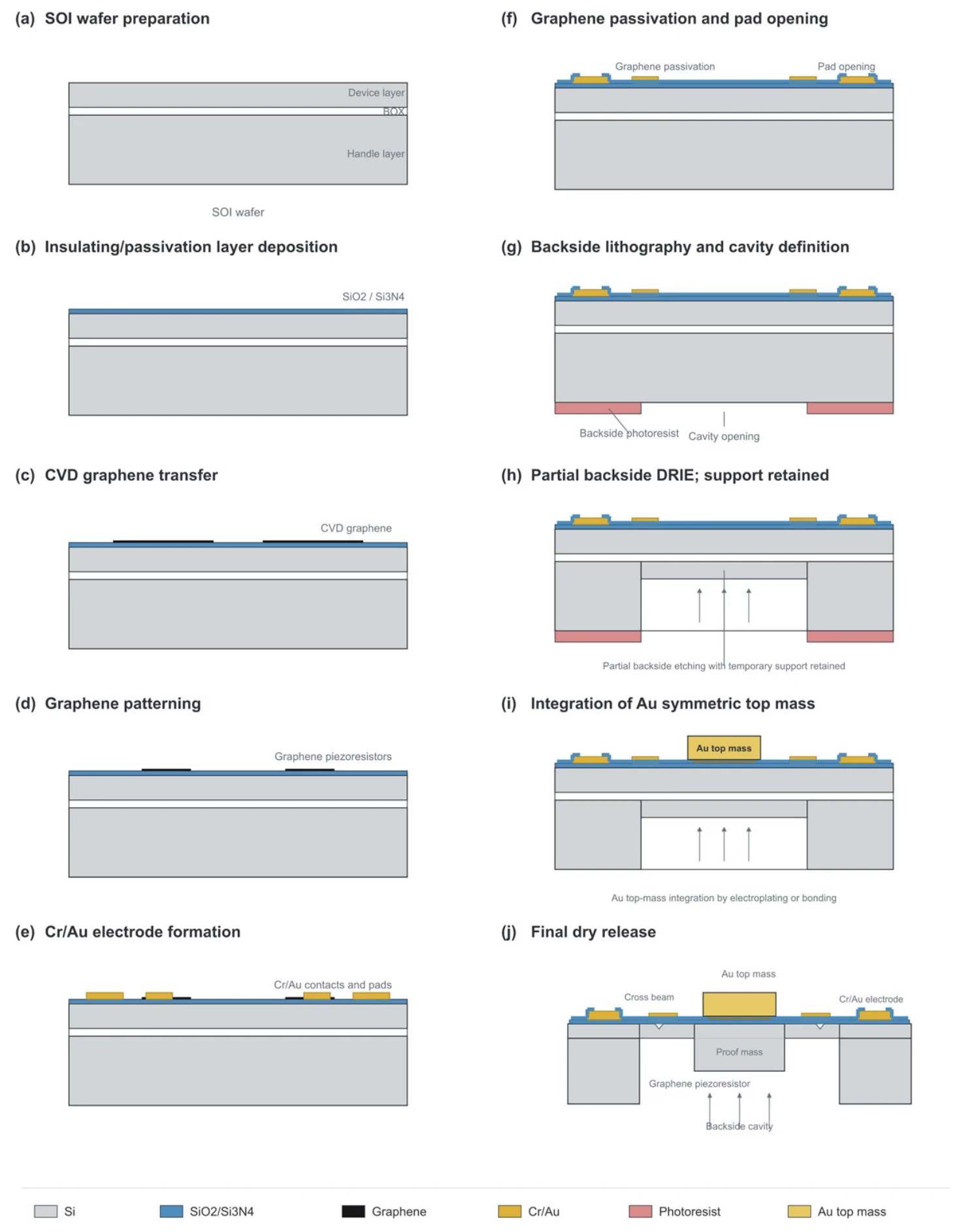
Conceptual fabrication process flow of the proposed high-g MEMS accelerometer with graphene piezoresistive readout. Graphene transfer, patterning, passivation, and Cr/Au contact formation are performed while the SOI wafer remains mechanically supported and approximately planar, followed by backside micromachining, Au symmetric top-mass integration, and final dry release.

Figure 11 summarizes this planar, support-retained fabrication sequence. Process-control structures may be included to monitor backside etch depth, temporary-support integrity, top-mass alignment, graphene continuity, and metal–graphene contact resistance before the final dry release.

Because the present work is a simulation-driven design study, direct experimental data are not yet available. To make this limitation explicit, the planned validation route includes fabricating reference chips with and without SCGs/symmetric masses, measuring graphene quality by Raman spectroscopy, extracting sheet/contact resistance by electrical test structures, calibrating the gauge factor under known strain, measuring the first-order resonant frequency, and performing high-g shock or Hopkinson-bar calibration to compare the measured bridge output with the predicted strain-to-voltage model.

In a fabricated device, the mismatch in the coefficients of thermal expansion among silicon, the SiO_2_/Si_3_N_4_ passivation layer, graphene, Cr/Au electrodes, and the Au top mass may generate residual and thermal stresses. These stresses may cause initial beam deformation, graphene pre-strain, zero-output drift, resonant-frequency shift, variation in strain-transfer efficiency, and interfacial delamination. These effects will be evaluated through coupled thermo-mechanical simulations and temperature-dependent experimental calibration in future work.

### 3.8. Mesh-Independence Verification

A mesh-independence study was performed using five mesh sizes of 5, 10, 15, 20, and 25 μm, with local refinement maintained near the beam roots and stress concentration grooves. The area-averaged strain in the graphene sensing region was used as the primary convergence indicator because it directly determines the piezoresistive output estimation. As shown in Table 4, for mesh sizes ranging from 5 to 20 μm, the area-averaged strain varied from 0.35936% to 0.36220%, corresponding to a maximum relative variation of approximately 0.79%. The maximum deflection also remained stable. The maximum equivalent stress showed larger fluctuations because it is more sensitive to local stress concentration near the groove-root region; therefore, it was treated as a supplementary reliability indicator rather than the primary convergence metric. Considering both numerical accuracy and computational efficiency, the 10 μm mesh was selected for the subsequent simulations.

## 4. Conclusions

This work presents the design of a graphene-based MEMS accelerometer for high-g measurements through a four-stage progressive structural optimization. Simulation results demonstrate that, compared with the initial cross-beam structure, the introduction of a proof mass enhances the peak strain at the beam root by approximately three-fold, while the first-order natural frequency decreases by only 7%. The subsequent integration of stress concentration grooves further augments the strain by approximately 8% over the beam-mass configuration, with a corresponding 8% decline in natural frequency. By incorporating symmetric masses, the cross-axis sensitivity is reduced by approximately 5.6% relative to the non-symmetric architecture. Ultimately, the finalized structure achieves a cumulative 15-fold increase in peak strain compared to the initial design. Although the first-order natural frequency decreases by approximately 55.7% from the baseline, it remains above 200 kHz. These findings indicate that the proposed progressive optimization scheme achieves an optimal trade-off between high sensitivity, cross-axis interference rejection, and dynamic response, providing a valuable reference for high-g piezoresistive MEMS accelerometers in aero-engine vibration monitoring. In the present analysis, the role of graphene is further clarified by introducing a surface piezoresistive transduction model. The area-averaged strain extracted from the graphene sensing regions was converted into resistance variation and bridge output voltage, providing a sensor-level electromechanical estimation rather than a purely mechanical structural report. It should be noted that the present work remains a simulation-based design study; graphene gauge factor, sheet resistance, contact resistance, fabrication tolerance, packaging stress, and high-g dynamic calibration should be experimentally verified in future work.

## Figures and Tables

**Table 1:** Cross-beam and groove dimensions.

Length (μm)	Width (μm)	Thickness (μm)	Groove Length (μm)
800	160	40	80

**Table 2:** Symmetric mass block dimensions.

Bottom Proof Mass Length (μm)	Bottom Proof Mass Thickness (μm)	Top Proof Mass Length (μm)	Top Proof Mass Thickness (μm)
500	140	400	70

**Table 3:** Quantitative comparison of reported high-g and graphene-based accelerometers with the proposed device.

Reference/ Device	Sensing Material/ Mechanism	Measurement Range (g)	Resonant Frequency (kHz)	Estimated Output at Stated Input	Validation Basis
Wung et al. [6]	Si piezoresistive; vertical-plate structure	Up to ≈3000 (quasi-static test)	—	≈9.00 mV/V_exc_ ^†^	Experimental; centrifugal rate-table test
Bae et al. [7]	Suspended Si piezoresistive bridges	2000	—	≈51.0 mV ^†^	Experimental; survived 4667 g
Dong et al. [9]	Axial-beam Si piezoresistive	2000	31	≈220 mV at 5 V ^†^	Experimental; survived 15,000 g
Wang et al. [4]	Biaxial Si piezoresistive	≤200,000 (FEA)	509.80/510.23 (X/Y)	≈278/284 mV at 5 V ^†^ (X/Y)	FEA + analytical estimation
Yu et al. [5]	Triaxial Si piezoresistive	9000–200,000 (tested)	1531/1524/1516 (X/Y/Z)	≈242.4/244.6/233.0 mV at 5 V ^†^ (X/Y/Z)	Experimental Hopkinson-bar tests; FEA
Jia et al. [34]	Self-support Si piezoresistive beams	100,000	445	≈54.0 mV/V_exc_ ^†^	FEA + experimental Hopkinson-bar test
Fan et al. [20]	Double-layer graphene ribbons + Si proof mass	Up to 2 (demonstrated; full-scale range not specified)	14.9–27.2	≈4.1 μV at 1 g and 100 μA ^†^	Electrical + shaker/LDV tests; FEA
This work	Graphene surface piezoresistors on Si beam-mass structure	250,000 (target)	>200 (simulated first mode)	4.53–22.64 mV at 5 V	FEA + analytical estimation (V_s_ = 5 V; GF = 2–10; R_c_ = 0.5R_g_)

^†^ Derived from data explicitly reported in the cited literature; these values were not reported directly as output measurements at the stated inputs. For Fan et al. [20], ≈4.1 μV is calculated from the reported resistance change of approximately 41 mΩ at 1 g using a measurement current of 100 μA. The source reports acceleration testing up to 2 g but does not specify a full-scale range. The proposed-device output is estimated at the 250,000 g target input for V_s_ = 5 V, GF = 2–10, and R_c_ = 0.5R_g_.

**Table 4:** Mesh-independence verification for the final accelerometer model.

Mesh Size (μm)	Area-Averaged Strain (%)	Maximum Deflection (μm)	Maximum Equivalent Stress (MPa)
5	0.35936	1.0976	642.30
10	0.36220	1.0972	607.11
15	0.35972	1.0956	685.91
20	0.36005	1.0897	570.40
25	0.36632	1.0874	662.77

## Data Availability

The data that support the findings of this study are available from the corresponding authors upon reasonable request.

## References

[1] Yang Y., Wang P., Chen M., Yang F., Liang Y., Chen F. (2022). A MEMS high-g acceleration sensor simulation analysis. J. Phys. Conf. Ser..

[2] Narasimhan V., Li H., Jianmin M. (2015). Micromachined high-g accelerometers: A review. J. Micromech. Microeng..

[3] Babatain W., Bhattacharjee S., Hussain A.M., Hussain M.M. (2021). Acceleration sensors: Sensing mechanisms, emerging fabrication strategies, materials, and applications. ACS Appl. Electron. Mater..

[4] Wang P., Yang Y., Chen M., Zhang C., Wang N., Yang F., Peng C., Han J., Dai Y. (2023). Design of a biaxial high-g piezoresistive accelerometer with a tension–compression structure. Micromachines.

[5] Yu M., Wu X., Zhao L., Jia C., Xia Y., Han X., Wang T., Luo G. (2025). Monolithically integrated triaxial high-performance high-g accelerometer for high shock vibration signal measurements. Microsyst. Nanoeng..

[6] Wung T.S., Ning Y.T., Chang K.H., Tang S., Tsai Y.X. (2015). Vertical-plate-type microaccelerometer with high linearity and low cross-axis sensitivity. Sens. Actuators A Phys..

[7] Bae K.M., Lee J.M., Kwon K.B., Han K.H., Kwon N.Y., Han J.S., Ko J.S. (2014). High-shock silicon accelerometer with suspended piezoresistive sensing bridges. J. Mech. Sci. Technol..

[8] Link B., Suminto J., Volk R. (1995). An Advanced, Monolithic Accelerometer for Safety Testing.

[9] Dong P.T., Li X.X., Wang Y.L., Feng S.L., Li S.Y. (2006). An axial-beam piezoresistive accelerometer for high-performance crash detection in the automotive industry. Proceedings of the 5th IEEE Conference on Sensors.

[10] Lee C., Wei X., Kysar J.W., Hone J. (2008). Measurement of the elastic properties and intrinsic strength of monolayer graphene. Science.

[11] Akinwande D., Brennan C.J., Bunch J.S., Egberts P., Felts J.R., Gao H., Huang R., Kim J.-S., Li T., Li Y. (2017). A review on mechanics and mechanical properties of 2D materials—Graphene and beyond. Extrem. Mech. Lett..

[12] Bolotin K.I., Sikes K.J., Jiang Z., Klima M., Fudenberg G., Hone J., Kim P., Stormer H. (2008). Ultrahigh electron mobility in suspended graphene. Solid State Commun..

[13] Zhu S.E., Ghatkesar M.K., Zhang C., Janssen G. (2013). Graphene-based piezoresistive pressure sensor. Appl. Phys. Lett..

[14] Khalid M.Y., Umer R., Zweiri Y.H., Kim J.-K. (2025). Rise of graphene in novel piezoresistive sensing applications: A review on recent development and prospect. Mater. Sci. Eng. R Rep..

[15] Wang J., Zhu Z., Qi Y., Li M. (2022). A novel crossbeam structure with graphene sensing element for N/MEMS mechanical sensors. Nanomaterials.

[16] Singh A., Lee S., Watanabe H., Lee H. (2020). Graphene-based ultrasensitive strain sensors. ACS Appl. Electron. Mater..

[17] Tang C., Wang Y., Li Y., Zeng S., Kong L., Li L., Sun J., Zhu M., Deng T. (2023). A review of graphene-based temperature sensors. Microelectron. Eng..

[18] Abrahams M.P., Martinez J., Steeneken P.G., Verbiest G.J. (2024). The graphene squeeze-film microphone. Nano Lett..

[19] Zhu Z., Wang J., Wu C., Chen X., Liu X., Li M. (2022). A wide-range and high-repeatability MEMS pressure sensor based on graphene. IEEE Sens. J..

[20] Fan X., Forsberg F., Smith A.D., Schröder S., Wagner S., Rödjegård H., Fischer A.C., Östling M., Lemme M.C., Niklaus F. (2019). Graphene ribbons with suspended masses as transducers in ultrasmall nanoelectromechanical accelerometers. Nat. Electron..

[21] Maharjan S., Samoei V.K., Jayatissa A.H. (2023). Graphene/PVDF nanocomposite-based accelerometer for detection of low vibrations. Materials.

[22] Ding J., Ma H., He C., Zhang W., Fan X. (2025). Two layers of carbon atoms enable ultrasensitive detection of acceleration. ACS Nano.

[23] Ding J., He C., Ma H., Zhang W., Fan X. (2025). Suspended graphene-based NEMS accelerometers with direct electrical readout. Microsyst. Nanoeng..

[24] Jin Z., Fang Z., Wang Z., Wu W., Yan D., Yang X., Gao Y., Yang W., Linxi D., Wang G. (2025). A high-performance NO2 gas sensor based on a silicon nanowire array. Phys. Scr..

[25] Dong C., Ye Y., Liu X., Yang Y., Guo W. (2019). The sensitivity design of a piezoresistive acceleration sensor in industrial IoT. IEEE Access.

[26] Oliva-Leyva M., Naumis G.G. (2015). Generalizing the Fermi velocity of strained graphene from uniform to nonuniform strain. Phys. Lett. A.

[27] Smith A.D., Niklaus F., Paussa A., Vaziri S., Fischer A.C., Sterner M., Forsberg F., Delin A., Esseni D., Palestri P. (2013). Electromechanical piezoresistive sensing in suspended graphene membranes. Nano Lett..

[28] Chen X., Zheng X., Kim J.-K., Li X., Lee D.-W. (2011). Investigation of graphene piezoresistors for use as strain gauge sensors. J. Vac. Sci. Technol. B.

[29] Shi Y., Jiang S., Liu Y., Wang Y., Qi P. (2022). Design and optimization of a triangular shear piezoelectric acceleration sensor for microseismic monitoring. Geofluids.

[30] Narzary T., Kumar R., Panwala F. (2018). MEMS-based diaphragm pressure sensor using S-shaped piezoresistors for enhancing sensitivity. Proceedings of the 2018 International Conference on Recent Trends in Electrical, Control and Communication.

[31] Tada K., Yasuda M., Kimoto Y., Kawata H., Hirai Y. (2008). Molecular dynamics study of yield stress of Si mold for nanoimprint lithography. Jpn. J. Appl. Phys..

[32] Jung H.I., Kwon D.S., Kim J. (2017). Fabrication and characterization of a monolithic piezoresistive high-g three-axis accelerometer. Micro Nano Syst. Lett..

[33] Zhao Y., Sun L., Liu Y., Wang W., Tian B. (2012). Incorporation of stress concentration slots into flexures for a high-performance microaccelerometer. Rev. Sci. Instrum..

[34] Jia C., Mao Q., Luo G., Zhao L., Lu D., Yang P., Yu M., Li C., Chang B., Jiang Z. (2020). Novel high-performance piezoresistive shock accelerometer for ultra-high-g measurement utilizing self-support sensing beams. Rev. Sci. Instrum..

